# Bacterial interfaces and surface chemistry shape hydroxyapatite mineral architecture during microbially induced calcium phosphate precipitation

**DOI:** 10.3389/fmicb.2026.1949480

**Published:** 2026-10-26

**Authors:** Pelina Toprak, Carlos Rodriguez-Navarro, Abhijit Mukherjee, Allan Pring, Navdeep K. Dhami

**Affiliations:** 1 School of Civil and Mechanical Engineering, Curtin University, Perth, WA, Australia; 2 Department of Mineralogy and Petrology, University of Granada, Granada, Spain; 3 Department of Earth Sciences, University of Adelaide, Adelaide, SA, Australia; 4 School of Molecular and Life Sciences, Curtin University, Perth, WA, Australia

**Keywords:** bacterial interfaces, biogenic mineral, biomineralization, calcium phosphate precipitation, extracellular polymeric substances, hydroxyapatite, microbe-mineral interactions, mineral phase evolution

## Abstract

Calcium phosphate biomineralization plays a crucial role in the formation of geological deposits, biological materials, and environmental remediation. While microorganisms are known to influence calcium phosphate precipitation, the relative contributions of bacterial cell surfaces and extracellular polymeric substances (EPS) remain poorly defined. This study presents the first microscale investigation of the distinct roles of Gram-positive and Gram-negative bacterial cells and EPS in regulating hydroxyapatite (HAp) mineralization, employing abiotic, EPS-mediated, and bacterial precipitation systems. Zeta potential measurements and Fourier Transform Infrared Spectroscopy (FTIR) revealed that both bacterial cells and isolated EPS provide negatively charged, chemically distinct interfaces capable of interacting with dissolved Ca^2+^ ions. Solution chemistry demonstrated greater phosphate and calcium ion depletion in biotic systems, consistent with enhanced crystal precipitation compared to abiotic controls. Field emission scanning electron microscopy (FESEM) and Brunauer–Emmett–Teller (BET) analyses showed that biological interfaces produced distinct crystal morphologies and surface characteristics. X-ray diffraction (XRD) revealed significant differences in phase composition: abiotic precipitation produced mixed calcium phosphate phases, whereas EPS-mediated precipitation promoted nucleation but remained dominated by precursor phases. In contrast, whole-cell systems favored HAp formation, with *Sporosarcina pasteurii* producing phase-pure crystalline HAp and *Pseudomonas fluorescens* producing HAp-dominant precipitates. Acid dissolution experiments further confirmed the increased stability of biogenic precipitates. These findings demonstrate that the spatial organization of bacterial surface functional groups, rather than surface charge alone, governs hydroxyapatite phase evolution, crystal growth, and mineral stability. While localized metabolic activity may contribute to microenvironmental conditions, the results highlight the importance of bacterial cell surfaces and EPS as key determinants of hydroxyapatite biomineralization, providing a mechanistic basis for the design of biologically mediated mineral materials.

## 1 Introduction

Microbially induced mineral precipitation (MIMP), also referred to as microbial biomineralization, is a widespread natural process driven by microbial activities that contributes to the formation of diverse geological structures in both terrestrial and marine environments, including travertines, coral systems and soil-associated mineral deposits (Murugan et al., 2021; Dhami et al., 2013). Through their metabolic activity and physicochemical interactions with surrounding fluids, microorganisms can promote the precipitation of a wide range of phosphate minerals, including calcium phosphate phases such as hydroxyapatite and brushite, magnesium ammonium phosphate phases such as struvite, and other biologically relevant phosphate minerals depending on microbial species and environmental conditions (Crosby and Bailey, 2012; Rivadeneyra et al., 2014). These processes are driven by microbial activities such as phosphate release through enzymatic hydrolysis, alterations in local pH and alkalinity, ammonium production, and the provision of reactive cell surfaces and extracellular polymeric substances (EPS), which concentrate ions and act as nucleation sites for mineral formation (Dong et al., 2022).

MIMP has also attracted interest for a range of engineering applications in civil and environmental engineering, including improving soil and concrete strength, repairing building materials, conservation of historical structures, immobilizing heavy metals and radionuclides, and enhanced oil recovery (Harkes et al., 2010; Burbank et al., 2011; Jroundi et al., 2017). To date, most applied research of biomineralization has focused on microbially induced carbonate precipitation (MICP) because of the versatility of CaCO₃-based biocementation systems (Whiffin et al., 2007). However, increasing attention has turned toward phosphate-based biominerals as a more stable alternative. Calcium phosphate phases, particularly hydroxyapatite (HAp), exhibit lower solubility and greater acid resistance than CaCO₃ and therefore have considerable potential as durable biocementing agents in environments where conventional MICP is ineffective (Qian et al., 2018; Cook, 1976; Glenn et al., 1994; Filippelli, 2011). Beyond geotechnical applications, HAp has also attracted increasing interest in environmental remediation, biomaterials, and bio-inspired mineral synthesis because of its chemical stability and biocompatibility (Guo et al., 2025; Eltarahony et al., 2025).

Many studies attribute microbial HAp formation primarily to enzymatic processes, particularly alkaline phosphatase-mediated phosphate release or the dissolution of organic phosphorus compounds (Cosmidis et al., 2015; Zorzetto et al., 2023). Recent work on microbial struvite precipitation demonstrated that differences in microbial phosphatase activity strongly influence phosphate mobilization and subsequent mineral precipitation (Toprak et al., 2025).

Thermodynamically, HAp can precipitate whenever sufficient Ca^2+^ and phosphate ions are present and solution conditions favor supersaturation:

$5{\mathrm{Ca}}^{2+}+3\mathrm{PO}{₄}^{3-}+{\mathrm{OH}}^{-}\to {\mathrm{Ca}}_{5}{\left({\mathrm{PO}}_{4}\right)}_{3}(\mathrm{OH})\;$ Thus, when dissolved phosphate is already abundant in solution, HAp precipitation can proceed without requiring microbial phosphate release as the primary phosphate source, indicating that microbial metabolism alone cannot fully explain the diversity of calcium phosphate phases, crystal morphologies, and mineral stabilities observed in biogenic systems. This suggests that, beyond controlling phosphate availability, microorganisms may also influence calcium phosphate mineralization through the physicochemical properties of their cell surfaces and extracellular matrices. Indeed, increasing evidence indicates that microbial interfaces themselves play an active role in regulating mineral nucleation and growth, thereby governing the spatial distribution, organization, and physicochemical properties of the resulting mineral phases (Dhami and Mukherjee, 2016; Li et al., 2015; Guo et al., 2025; Toprak et al., 2025).

Bacterial cell surfaces and EPS provide chemically distinct microenvironments enriched in negatively charged functional groups, including carboxyl, phosphoryl, and hydroxyl moieties (Figure 1). These groups bind Ca^2+^ and locally concentrate reactants, thereby altering local ion availability and supersaturation and influencing nucleation pathways at the nano- to microscale (Dubey et al., 2025; Baker et al., 2010). However, bacterial cells and EPS differ fundamentally in their structural organization and surface chemistry, and therefore impose different constraints on ion binding, nucleation density, and subsequent crystal growth (Decho, 2010; Fein et al., 1997; Qu et al., 2022). Bacterial cells are enclosed by a structurally complex cell envelope that, despite variability among species, universally contains a peptidoglycan matrix composed of cross-linked glycan chains interconnected by peptide bridges (Brown et al., 2013). In Gram-positive (G+) bacteria, such as *Sporosarcina pasteurii*, this matrix is substantially thickened and densely functionalized with anionic glycopolymers, particularly wall teichoic acids, resulting in highly negatively charged and structurally rigid interfaces (Neuhaus and Baddiley, 2003). These characteristics enhance cation binding and favor localized nucleation, generating localized supersaturation conditions (Hoffmann et al., 2021). In contrast, Gram-negative (G−) bacteria, such as *Pseudomonas fluorescens*, display lipopolysaccharide-rich outer membranes, presenting more dynamic and chemically heterogeneous interfaces with comparatively lower structural rigidity (Nikaido, 2003). This structural organization leads to more diffuse ion distributions and less spatially constrained nucleation, promoting more spatially dispersed mineral nucleation and reduced control over crystal growth compared with G + systems (Hoffmann et al., 2021).

**Figure 1:**
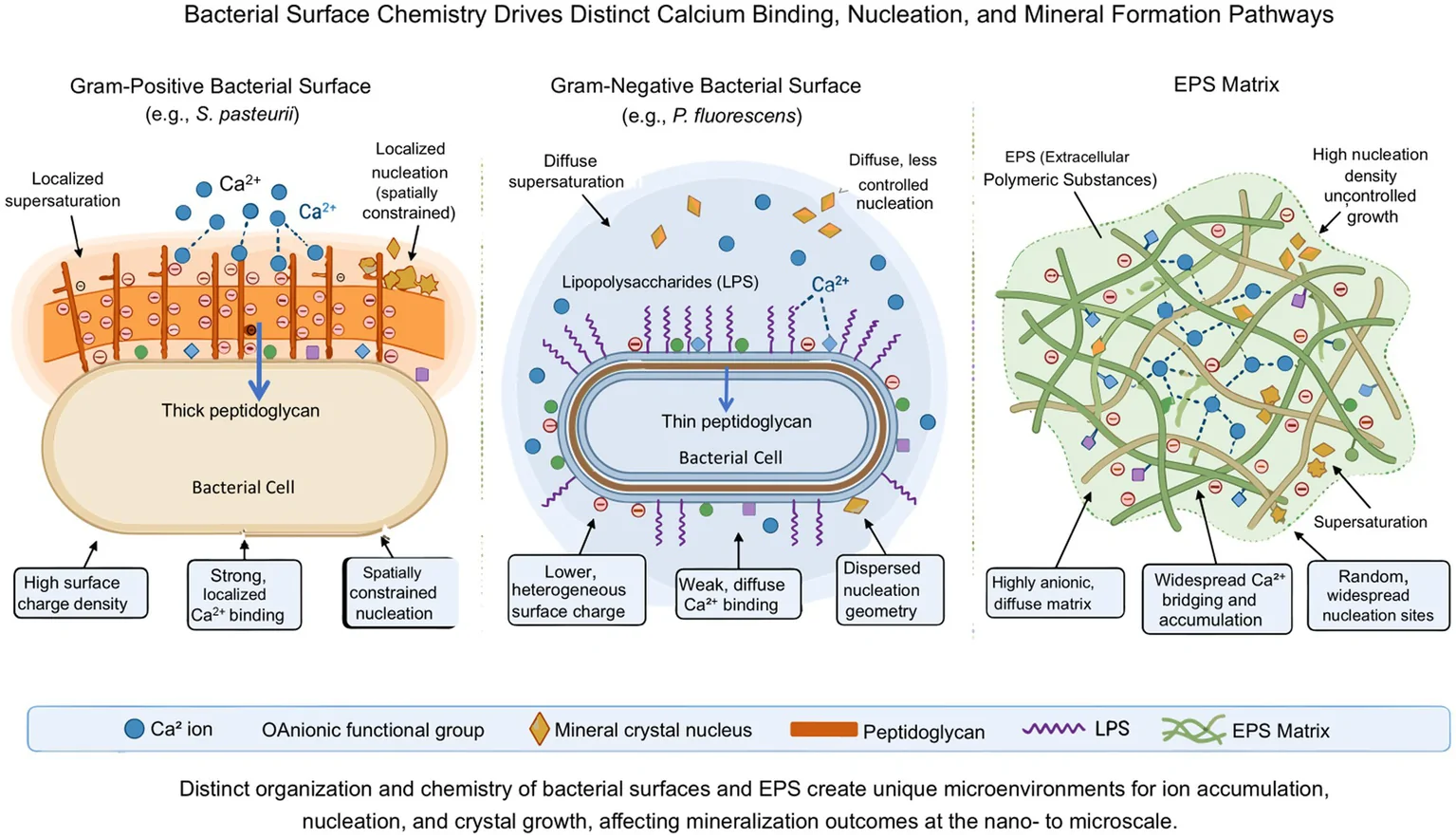
Distinct bacterial surfaces and EPS matrices drive different Ca^2+^ binding, nucleation, and phosphate mineralization pathways. Created in BioRender. (Toprak) Polat, P. (2026) https://BioRender.com/rtpy03k.

In addition to the cell envelope, many bacteria produce EPS, which are complex hydrated matrices composed of proteins, polysaccharides, lipids, and extracellular DNA (Flemming and Wingender, 2010; Dubey et al., 2025). Unlike the relatively rigid and spatially fixed interfaces of bacterial cell surfaces, EPS form porous, chemically heterogeneous networks rich in negatively charged functional groups, including carboxyl, phosphoryl, and hydroxyl moieties, which impose additional interfacial control over mineral formation (Decho, 2010; O’Toole et al., 2000). In bacterially induced CaCO₃ precipitation, such EPS-rich environments have been shown to stabilize metastable vaterite, whereas low-EPS systems favor transformation to calcite (Dubey et al., 2025). Consequently, EPS promote crystal growth regimes distinct from those associated with cell-bound interfaces, often delaying crystallization and favoring the formation of nanocrystalline or defect-rich phases with distinct physicochemical properties (Ercole et al., 2007; Flemming and Wingender, 2010; Toprak et al., 2025).

Despite these advances, the relative contributions of intact bacterial cells and EPS as independent drivers of mineral nucleation remain poorly understood, largely because most studies do not separate metabolic activity from interfacial effects (Dong et al., 2022; Wang et al., 2020; Qin et al., 2020). Although a small number of studies in MICP have begun to distinguish between the effects of whole cells and EPS on carbonate mineralization (Dubey et al., 2025), comparable investigations for calcium phosphate systems remain lacking. Consequently, it remains unclear to what extent hydroxyapatite formation is governed by biochemical phosphate generation versus physicochemical templating and confinement at biologically derived interfaces, particularly under conditions where phosphate is already readily available in solution (Decho, 2010; Guo et al., 2025). A critical mechanistic question therefore arises in systems where phosphate is already available in soluble form. Such conditions are commonly encountered in phosphate-rich waters affected by fertilizers or sewage (eutrophic systems), as well as in natural sedimentary phosphate deposits (Nadagouda et al., 2024; Föllmi, 1996). Understanding how microorganisms influence mineralization in these settings is of direct environmental significance, as bacterially mediated precipitation of highly insoluble hydroxyapatite offers a promising pathway for the removal and recovery of excess phosphate from contaminated waters (Nadagouda et al., 2024). Under such conditions, hydroxyapatite precipitation may proceed predominantly through physicochemical pathways once supersaturation is reached, reducing the requirement for microbial phosphate release (Poorhemati and Komarova, 2023; He et al., 2020). In these environments, the principal role of microorganisms may shift from phosphate generation toward the regulation of nucleation pathways, precursor stabilization, crystal morphology, and mineral stability through their interfacial properties. However, the relative contributions of bacterial interfacial properties and localized metabolic processes to calcium phosphate mineralization remain incompletely resolved.

To address this question, we established a comparative experimental framework designed to evaluate the influence of bacterial interfaces on calcium phosphate precipitation under conditions where phosphate was already present in soluble form. Purely abiotic precipitation was compared with systems mediated by intact bacterial cells and EPS. Two model microorganisms representing distinct cell envelope architectures were selected: the G + bacterium *S. pasteurii* and the G − bacterium *P. fluorescens*. In addition, purified EPS extracted from *P. fluorescens* cultures was used to distinguish the effects of extracellular polymers from those of intact cells.

By integrating zeta potential measurements, precipitation kinetics, mineral phase analysis, surface area characterization, and acid dissolution experiments, this study directly links bacterial interfacial properties to the formation and stability of hydroxyapatite. This approach provides a mechanistic evaluation of how bacterial cells and EPS influence calcium phosphate biomineralization beyond bulk solution chemistry and demonstrates that microbial interfaces act not merely as passive substrates but as active determinants of mineral architecture, crystallization pathways, and stability.

## 2 Materials and methods

### 2.1 Hydroxyapatite precipitation: experimental design

To evaluate the contribution of distinct bacterial interfaces to calcium phosphate mineralization, four experimental systems were established:

#### 2.1.1 *Sporosarcina pasteurii*-mediated precipitation

*S. pasteurii* (ATCC 11859), a G+, rod-shaped soil bacterium characterized by a thick peptidoglycan cell wall (Wu et al., 2021) and relatively low EPS production (Dubey et al., 2025), was cultivated in ammonium yeast extract (YAT) medium at 37 °C and 120 rpm following the method of Stocks-Fischer et al. (1999). Cultures were harvested at the mid-exponential phase (OD₆₀₀ ≈ 0.5), centrifuged, and washed with sterile phosphate-free buffer to remove residual medium components that could influence mineral supersaturation.

Standardized cell suspensions were introduced into a calcium phosphate precipitation medium adapted from established hydroxyapatite synthesis protocols (Koutsopoulos, 2002; Dorozhkin, 2007). The medium contained 2 g L^−1^ yeast extract, 10 mM KH₂PO₄, and 16 mM CaCl₂ (Ca/*P* = 1.6) and was adjusted to pH 8.0 prior to inoculation.

#### 2.1.2 *Pseudomonas fluorescens*-mediated precipitation

*P. fluorescens* (ATCC 13525), a G − bacterium characterized by an outer membrane rich in lipopolysaccharides and typically producing substantial quantities of EPS (Vesper, 1987; Oliveira et al., 1994), was cultivated in Luria-Bertani (LB) broth at 37 °C and 120 rpm.

Cells were harvested at the mid-exponential phase (OD₆₀₀ ≈ 0.5), centrifuged, and washed with sterile phosphate-free buffer to eliminate residual growth medium components. Standardized cell suspensions were transferred into the same calcium phosphate precipitation medium described above (2 g L^−1^ yeast extract, 10 mM KH₂PO₄, 16 mM CaCl₂; Ca/*P* = 1.6, pH 8.0).

#### 2.1.3 Precipitation mediated by an isolated extracellular polymeric substance fraction

EPS were isolated from *P. fluorescens* cultures following a modified ethanol precipitation method based on Bains et al. (2015). Briefly, bacterial cultures were grown in 100 mL of Luria-Bertani (LB) broth at 37 °C for 48 h. Following incubation, cultures were centrifuged at 13,200 × g for 25 min at 4 °C to remove intact cells and cellular debris. The resulting cell-free supernatant was collected for EPS isolation. EPS was precipitated from 50 mL of supernatant by the addition of three volumes of chilled absolute ethanol and incubated at 4 °C overnight. The precipitated material was recovered by centrifugation (13,200 × g, 15 min, 4 °C) and air-dried at room temperature. The isolated EPS fraction was introduced into the same precipitation medium as described in sections 2.1.1 and 2.1.2 in the absence of intact cells to evaluate its behavior as a cell-free polymeric nucleation interface.

The EPS treatment was included as a cell-free polymeric interface to compare its mineralization behavior with that of intact bacterial cell surfaces. As EPS was isolated only from 48 h *P. fluorescens* cultures grown in LB, this treatment represents a model EPS interface rather than a species-matched comparison or EPS produced directly under mineralizing conditions.

#### 2.1.4 Abiotic precipitation

An abiotic control system containing the same precipitation medium (2 g L^−1^ yeast extract, 10 mM KH₂PO₄, 16 mM CaCl₂; pH 8.0) was prepared without cells or EPS to evaluate purely chemical calcium phosphate precipitation under identical conditions.

#### 2.1.5 Non-viable-cell comparison

To assess whether continued cellular viability was required for mineral formation, supplementary mineralization assays were conducted using paraformaldehyde (PFA)-fixed non-viable cells of *S. pasteurii* and *P. fluorescens*. Loss of viability was verified on the corresponding growth media prior to mineralization. The fixed-cell assays were conducted in triplicate (n = 3) under the same mineralization conditions as the corresponding live-cell treatments. Preparation of the PFA-fixed cells and the subsequent pH, FESEM and qualitative XRD analyses are described in Supplementary Information (section S1; Supplementary Figure S1).

### 2.2 Incubation and sampling

Mineralization assays were performed in 250 mL Erlenmeyer flasks containing 100 mL of precipitation medium and incubated at 37 °C with orbital shaking (120 rpm) for 5 days. All mineralization assays were conducted using three independent biological replicates.

At 24 h intervals throughout the five-day incubation period, 2 mL aliquots of the medium were withdrawn aseptically. Sample pH was measured before filtration using a calibrated pH meter to monitor changes in bulk solution chemistry during mineralization. The samples were subsequently passed through sterile 0.2 μm membrane filters to remove bacterial cells and suspended mineral particles. The filtered supernatants were stored at 4 °C until chemical analysis.

Dissolved inorganic phosphate (Pi) was measured using a modified ascorbic acid–molybdate colorimetric assay based on Chen et al. (1956). Under acidic conditions, orthophosphate was complexed with ammonium molybdate in the presence of potassium antimony tartrate. The resulting phosphomolybdate complex was reduced with ascorbic acid to generate a blue-colored product. Its absorbance was recorded at 880 nm, and Pi concentrations were obtained by comparison with a calibration curve prepared using known phosphate standards.

Residual dissolved Ca^2+^ in the filtered supernatants was quantified by EDTA complexometric titration according to Standard Methods (APHA, 2012). A 40 μL aliquot of each supernatant was diluted to a final volume of 10 mL with deionized water. The solution pH was then increased by adding 400 μL of 1 N NaOH, followed by several drops of hydroxy naphthol blue disodium salt indicator (1% w/v). Each prepared sample was titrated with 1 mM disodium EDTA until a stable color transition from pink to blue was observed. CaCl₂ standards spanning 0–20 mM, prepared in the same precipitation medium as the samples, were carried through the identical dilution and titration procedure. A calibration curve curve was generated by relating the Ca^2+^ concentration of each standard to the corresponding EDTA volume required to reach the endpoint. Unknown Ca^2+^ concentrations in the supernatants were determined from the resulting calibration relationship.

### 2.3 Crystal harvesting

After 5 days of incubation, mineral precipitates were collected by filtration using 0.45 μm Whatman membrane filters. The recovered precipitates were dried at 37 °C until a constant mass was achieved and then weighed directly using an analytical balance. Mineral yield was calculated by normalizing the dry precipitate mass to the initial culture volume and was expressed as g L^−1^. The recovered solids were subsequently used for morphological, mineralogical and chemical characterization. Because a single 100 mL incubation yields a limited quantity of solid, material for surface area and acid dissolution analyses was obtained by pooling precipitate recovered from independent incubations per system, carried out under identical conditions to those described above. Pooled material was homogenized by gentle grinding prior to subsampling.

### 2.4 Interfacial characterization (cells and EPS)

For interfacial characterization, *S. pasteurii* and *P. fluorescens* were cultivated under their respective species-specific growth conditions described in section 2.1. Cultures were harvested at mid-exponential phase (OD₆₀₀ ≈ 0.5) to provide cells at a comparable physiological growth stage and to minimize variability associated with stationary-phase surface restructuring (Halder et al., 2015).

Cells were collected by centrifugation (3,000 × g, 5 min, 25 °C) and washed twice with Milli-Q water to remove residual growth-medium components and loosely associated ions that could interfere with surface charge determination. Washed cells were subsequently resuspended in 0.5 mM potassium chloride (KCl) adjusted to pH 7.4 (Soon et al., 2011). For zeta-potential analysis of EPS, isolated EPS fractions were dispersed in the same electrolyte solution (0.5 mM KCl, pH 7.4) and gently homogenized to ensure uniform dispersion prior to analysis.

Zeta potential is influenced by both intrinsic surface chemistry and the physicochemical properties of the surrounding medium. Ionic strength and pH influence ion adsorption and the ionization state of surface functional groups, thereby affecting electrophoretic mobility (Takashima and Morisaki, 1997; Soon et al., 2011). To provide a common analytical environment and minimize differences arising from ionic screening, all bacterial-cell and EPS measurements were therefore conducted in 0.5 mM KCl at pH 7.4.

Zeta potential was determined using a Zetasizer Nano ZS 90 (Malvern, United Kingdom) at room temperature. Each condition was analyzed using three independently prepared biological replicates (*n* = 3).

Surface-associated functional groups of the bacterial cells and isolated EPS were characterized using Fourier transform infrared (FTIR) spectroscopy (Bruker IFS66). Spectra were acquired in absorbance mode over the range 400–4,000 cm^−1^ using 20 consecutive scans at a spectral resolution of 4 cm^−1^.

### 2.5 Mineral phase identification

Phase composition and crystallographic characteristics of the obtained mineral precipitates were determined by X-ray diffraction (XRD) analysis. Finely ground samples were suspended in ethanol and transferred onto low-background sample holders for measurement.

Diffraction patterns were recorded using a Bruker D8 Advance diffractometer equipped with Ni-filtered Cu Kα radiation (λ = 1.5406 Å), operated at 40 kV and 40 mA. Data were collected across a 2θ range of 7–120° with a step size of 0.015°.

Phase identification was carried out using the XPowder software package, with reference patterns obtained from the International Centre for Diffraction Data (ICDD) database. Identified crystalline phases were matched based on characteristic peak positions and intensities. Semi-quantitative phase analysis was performed using the Reference Intensity Ratio (RIR) method, as implemented within XPowder. Relative phase proportions were calculated using the corresponding ICDD reference files for each identified phase. Reported phase proportions represent the relative abundances of the identified crystalline phases and therefore do not account for any amorphous calcium phosphate that may have been present.

### 2.6 Morphology and microstructure characterization

Morphology and microstructure of the recovered precipitates were characterized by field-emission scanning electron microscopy (FESEM) (MIRA3, TESCAN). Prior to observation, samples were fixed onto aluminum mounts using conductive carbon tape and sputter-coated with a thin platinum layer to minimize charging during imaging.

### 2.7 Surface area and pore structure analysis

The specific surface area of the recovered precipitates was determined by nitrogen adsorption–desorption analysis using a Micromeritics TriStar II Plus (v3.04; Micromeritics Instrument Corporation, Norcross, GA, United States). Nitrogen (N₂) was used as the adsorbate at 77.15 K (liquid nitrogen temperature). The relative pressure (P/P₀) range was controlled automatically by the instrument, with the reference pressure defined as P₀ = 101.325 kPa under standard operating conditions.

Approximately 1 g of pooled precipitate was accurately weighed and loaded into the analysis tube. Prior to measurement, samples were degassed at 60 °C under helium flow to remove adsorbed moisture, CO₂, and other weakly bound gaseous species. After degassing, each tube was reweighed to determine the precise dry mass used for analysis.

Adsorption–desorption isotherms were acquired using a predefined sequence of 60 equilibrium P/P₀ points spanning approximately 0.01–0.99. The adsorption branch was measured with stepwise increases in relative pressure, followed by the desorption branch using stepwise pressure reductions.

The specific surface area was calculated from the adsorption data using the Brunauer–Emmett–Teller (BET) method within the linear BET region (typically P/P₀ ≈ 0.05–0.30). Pore size distribution, cumulative pore volume, and cumulative pore surface area were determined by the Barrett–Joyner–Halenda (BJH) method applied to the desorption branch of the isotherm, over a pore width range of 17–3,000 Å. Micropore surface area and micropore volume were assessed using the t-Plot method, with a negative or near-zero micropore volume interpreted as the absence of meaningful microporosity. All data reduction and calculations were performed using the TriStar II Plus Version 3.04 software (Micromeritics Instrument Corporation, Norcross, GA, United States).

BET and BJH analyses were performed on one representative pooled precipitate from each experimental system as material characterization measurements. Pooling produced a composite sample representing material recovered from the pooled incubations, but it did not provide analytical replication or an estimate of between-incubation variability. The BET and BJH values are therefore presented as single descriptive measurements, are not used for inferential statistical comparison, and were interpreted together with the XRD phase assemblages and FESEM observations to distinguish the effects of mineralogy from those of surface development.

### 2.8 Acid dissolution experiment

The acid stability of precipitated hydroxyapatite (HAp) was evaluated using a controlled batch dissolution assay under mildly acidic conditions (pH 4.0). The experimental design was adapted from classical hydroxyapatite dissolution studies (Budz and Nancollas, 1988), with modifications appropriate for comparative evaluation of abiotic and microbially mediated HAp systems.

Dissolution was conducted in 0.1 M sodium acetate–acetic acid buffer adjusted to pH 4.00 ± 0.02 with a calibrated pH meter. For each precipitation system, 50.0 ± 0.1 mg of dried precipitate was suspended in 50.0 mL of the buffer in acid-resistant polypropylene tubes and held under static batch conditions without replacement of the dissolution medium, with pH maintained by the buffer rather than by acid addition. (A separate tube was prepared for each sampling time.) The pH of each suspension was verified at every sampling interval and remained within pH 3.90–4.10 throughout the 12 h period. At predetermined time intervals (2, 6 and 12 h), suspensions were vacuum filtered using pre-weighed membrane filters. The recovered solids were briefly rinsed with ultrapure water to remove residual acidic solution buffer and dried at 60 °C to constant mass. Dissolution was quantified as the decrease in remaining dry mass over time. For cross-system comparison, results were expressed as remaining solid mass (mg) relative to the initial mass. For the two HAp-rich biogenic precipitates, an additional surface-area-normalized dissolution rate was calculated using BET surface area values.

### 2.9 Statistical analysis

Quantitative data obtained from independently replicated biological experiments are presented as mean ± standard deviation (SD). Mineralization assays, calcium and phosphate ion measurements, pH measurements and acid dissolution experiments were each performed using three independent biological replicates, unless otherwise stated.

Material characterization techniques, including XRD, FESEM, and BET/BJH analyses, were performed on representative precipitates from each experimental system and are presented descriptively. These characterization data were interpreted in conjunction with one another to evaluate differences in mineral phase composition, morphology, surface area, and pore structure.

## 3 Results

### 3.1 Interfacial properties of cells and EPS

Zeta potential measurements revealed clear differences in surface charge between whole bacterial cells and isolated EPS (Figure 2a). All exhibited net negative zeta potentials. *S. pasteurii* displayed moderately negative values (approximately −25 mV), whereas *P. fluorescens* exhibited a substantially more negative surface charge (approximately −38 mV). The isolated EPS fraction showed the most negative zeta potential (approximately −49 mV), indicating a highly anionic interfacial environment. These differences likely reflect variations in interfacial chemistry between G + and G − cell envelopes, as well as the enrichment of ionizable functional groups within the extracellular matrix. The strongly negative zeta potential of EPS suggests the presence of a high density of negatively charged functional groups capable of electrostatic interaction with dissolved divalent cations.

**Figure 2:**
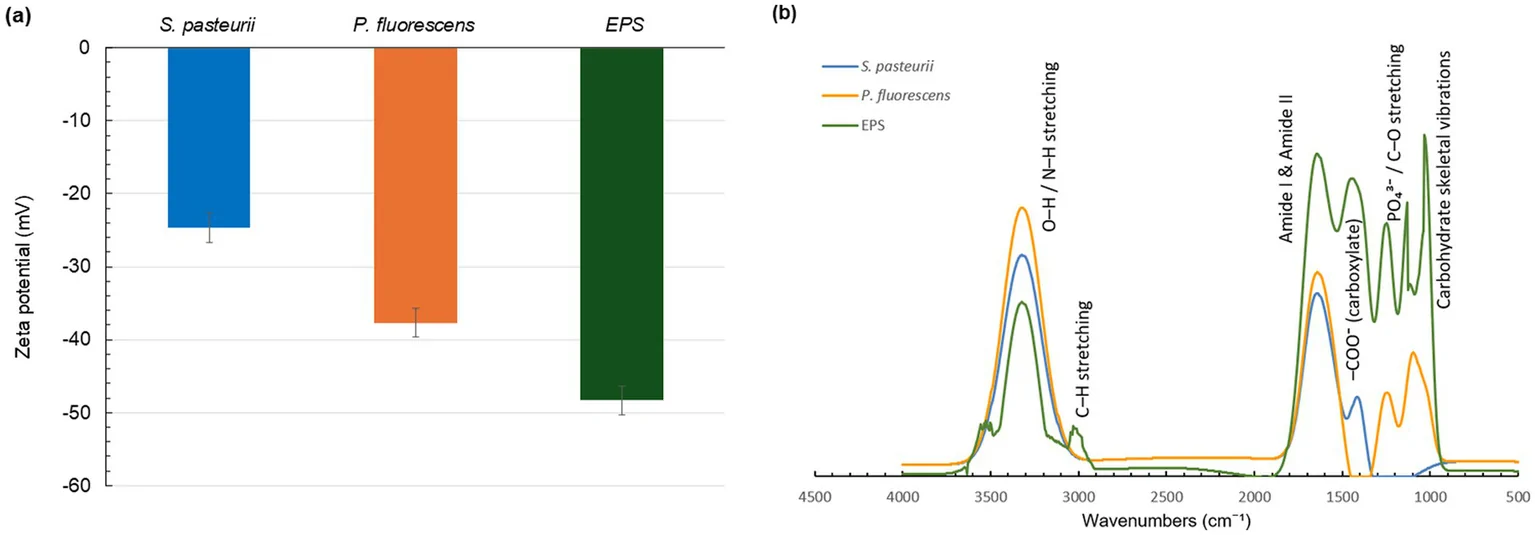
**(a)** Zeta potential measurements of *S. pasteurii*, *P. fluorescens*, and EPS. **(b)** FTIR spectra highlighting characteristic functional groups. Data in **(a)** are presented as mean ± SD (*n* = 3).

Measurements were conducted in 0.5 mM KCl at pH 7.4 (section 2.4) and therefore describe the intrinsic surface charge of each interface rather than its electrokinetic state within the precipitation medium, in which 16 mM Ca^2+^ will compress the electrical double layer and screen these potentials.

FTIR spectroscopy was performed to identify the surface functional groups that may contribute to the differences in interfacial charge observed in the zeta potential measurements (Figure 2b). Cells of both bacterial species exhibited broad absorption bands in the 3,200–3,500 cm^−1^ region, corresponding to overlapping O–H and N–H stretching vibrations associated with hydroxyl-containing polysaccharides, proteins, and hydration water. Distinct amide I (~1,650 cm^−1^) and amide II (~1,540 cm^−1^) bands were observed in both whole-cell and EPS spectra, indicating the presence of proteins within the interfacial matrix. Carboxylate-associated vibrations (–COO^−^) were evident in the 1,400–1,450 cm^−1^ region, suggesting the presence of acidic functional groups derived from amino acids and acidic polysaccharides. These negatively charged functional groups are well known to act as metal-binding sites and likely contribute to the negative surface potentials observed in the zeta potential analysis. In addition, the fingerprint region (900–1,200 cm^−1^) displayed enhanced intensity and spectral complexity in the EPS fraction compared to whole cells (Figure 2b). These bands correspond to phosphate (PO2^−^) and carbohydrate skeletal vibrations, consistent with the enrichment of phosphorylated biomolecules and polysaccharides within the extracellular matrix. A band in the 2,850–2,950 cm^−1^ region, assigned to aliphatic C–H stretching vibrations, was observed more prominently in the EPS spectrum, indicating the presence of aliphatic and/or hydrophobic molecular components that were less evident in intact cell spectra.

Collectively, the electrokinetic measurements (Figure 2a) and FTIR functional-group analysis (Figure 2b) show that intact bacterial cells and isolated EPS provide chemically distinct, negatively charged interfaces capable of interacting with dissolved ions. Among the interfaces examined, the isolated EPS fraction exhibited the most negative zeta potential and pronounced contributions from ionisable functional groups. These observations are consistent with a capacity for cation association at the EPS interface; however, surface charge alone did not predict subsequent mineral phase composition. These findings apply specifically to the isolated *P. fluorescens*-derived EPS fraction examined in this study.

### 3.2 Solution chemistry during mineralization and mineral yield

Gravimetric analysis after 5 days revealed clear differences in total recovered solid mass among the systems (Figure 3a). The *P. fluorescens* system produced the highest dry weight at 1.97 ± 0.10 g L^−1^, followed by *S. pasteurii* at 1.80 ± 0.09 g L^−1^. The EPS-mediated system yielded 1.49 ± 0.06 g L^−1^ and the abiotic control the lowest at 0.84 ± 0.06 g L^−1^. Values for the whole-cell and EPS-mediated systems represent total recovered solid and therefore include bacterial biomass or added polymer retained on the filter alongside the mineral phase; they are not directly comparable to the abiotic value on a mineral-mass basis.

**Figure 3:**
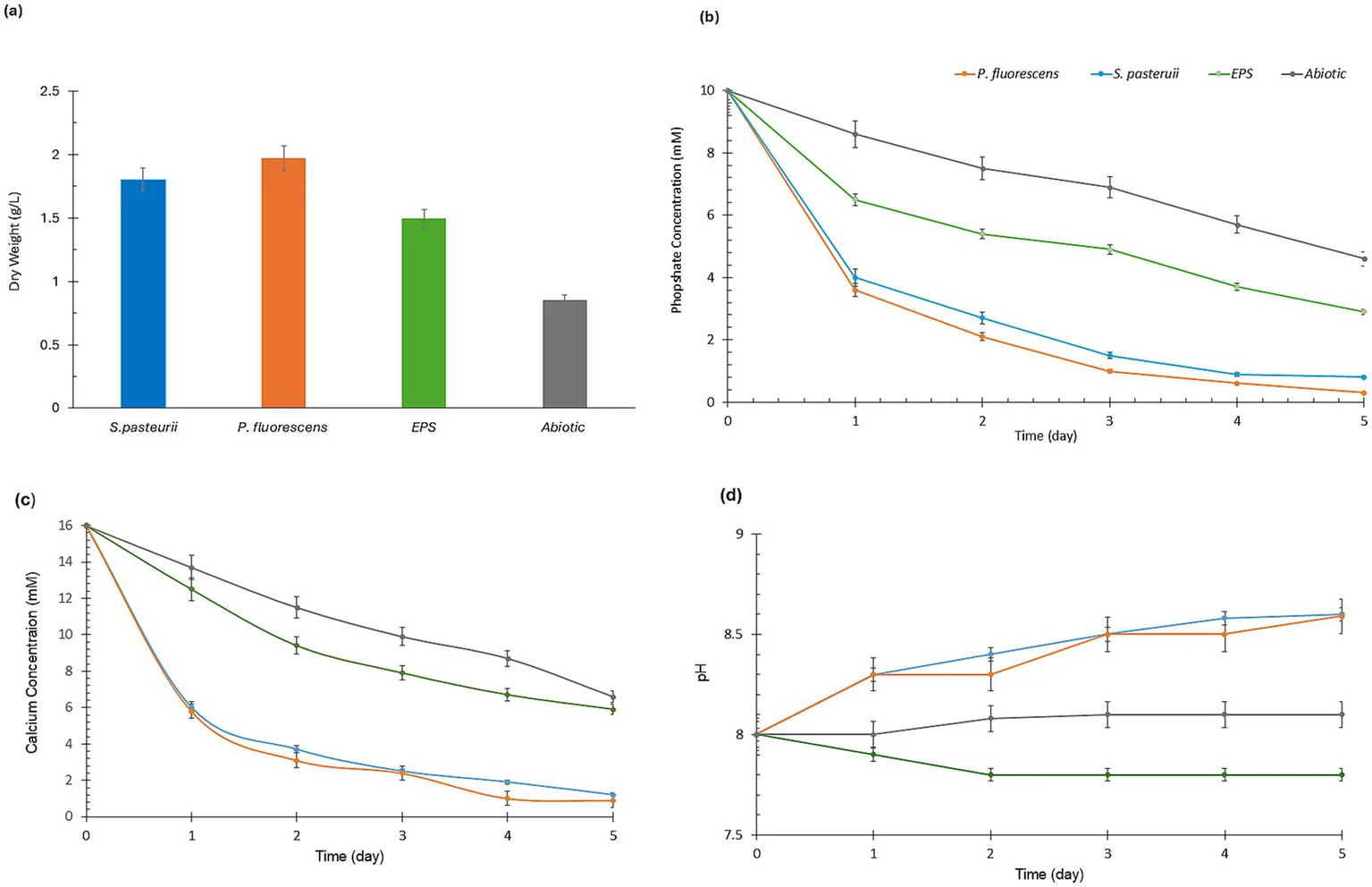
Precipitation kinetics and mineral yield during calcium phosphate formation in biogenic and abiotic systems. **(a)** Dry mineral mass recovered after 5 days of incubation. **(b)** Soluble phosphate concentration over time, **(c)** Soluble calcium (Ca^2+^) concentration over time. **(d)** Solution pH evolution during incubation. Data represent mean ± SD from three independent experiments (*n* = 3).

Soluble phosphate and calcium (Ca^2+^) concentrations, together with pH, were monitored during incubation to evaluate ion depletion associated with calcium phosphate precipitation across all systems (Figures 3b–d), using the colorimetric and complexometric procedures described in section 2.2.

Dissolved phosphate concentrations decreased progressively over the 5-day incubation period in all systems (Figure 3b), indicating continuous incorporation of phosphate into the precipitated mineral phase. Biologically mediated systems exhibited more rapid phosphate depletion compared with the abiotic control. The *P. fluorescens* system showed the fastest decline, with phosphate falling from 10.0 to 3.6 mM within the first 24 h (64% removal) and reaching 0.6 mM by day 5 (94% removal). The *S. pasteurii* system displayed a similar but slightly slower trend, declining to 4.1 mM at day 1 and 0.8 mM at day 5 (92% removal). The EPS-mediated system exhibited intermediate behavior, decreasing more gradually to 6.5 mM at day 1 and 2.9 mM at day 5 (71% removal). The abiotic system showed the slowest depletion, retaining 8.6 mM at day 1 and 4.6 mM at day 5, corresponding to 54% removal over the full incubation.

Dissolved calcium concentrations followed a similar trend (Figure 3c), showing a monotonic decline consistent with progressive calcium phosphate precipitation. The most rapid Ca^2+^ removal occurred in the biologically mediated systems, in which concentrations fell from 16.0 mM to approximately 6.0 mM within the first 24 h and reached 0.9 mM (*P. fluorescens*) and 1.2 mM (*S. pasteurii*) by day 5, corresponding to 94 and 93% removal, respectively. Calcium depletion in the EPS system was slower, reaching 5.8 mM by day 5 (64% removal), and the abiotic control exhibited gradual removal to 6.5 mM (59%) over the full 5-day period. Relative to the abiotic control, both intact-cell treatments and the EPS treatment exhibited greater Ca^2+^ depletion, consistent with enhanced calcium incorporation into the recovered precipitate under the conditions tested.

Solution pH exhibited modest but system-dependent changes during incubation (Figure 3d). The pH of the *S. pasteurii* and *P. fluorescens* treatments increased from 8.0 to 8.3 within the first 24 h and thereafter to 8.58–8.60 by day 5. In contrast, the pH of the EPS treatment decreased to approximately 7.8 by day 2 and remained constant thereafter, while the abiotic control remained close to pH 8.0 with only minor fluctuations. These values represent the net pH response of each system, potentially reflecting contributions from calcium phosphate precipitation, aqueous phosphate buffering, proton exchange with EPS functional groups and, in the whole-cell treatments, residual metabolic activity.

### 3.3 Phase composition of calcium phosphate precipitates

X-ray diffraction (XRD) analysis revealed distinct phase compositions across the four experimental systems (Figure 4; Table 1). The abiotic control produced a multiphase calcium phosphate assemblage comprising apatite-type calcium phosphate (61 wt%), brushite (13 wt%), and monetite (26 wt%) (Figure 4a). The apatite contribution was initially fitted as hydroxyapatite (27 wt%) and chlorapatite (34 wt%). However, because hydroxyapatite and chlorapatite are structurally closely related apatite endmembers with strongly overlapping diffraction patterns, the two components could not be reliably resolved as independently crystallized phases from the present XRD data. They were therefore combined and are reported collectively as apatite-type calcium phosphate (61 wt%).

**Figure 4:**
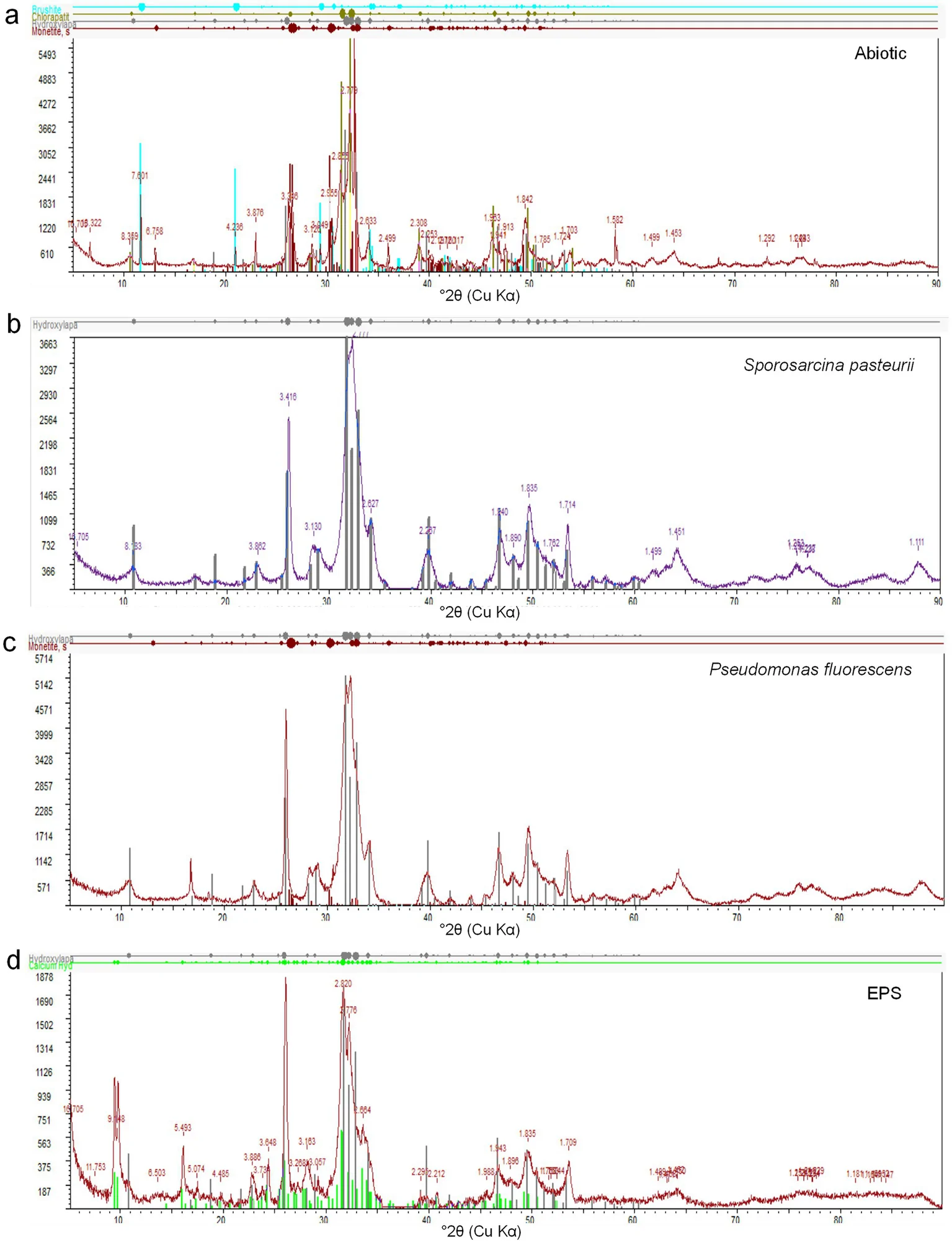
XRD patterns of calcium phosphate precipitates formed under different experimental systems: **(a)** abiotic chemical precipitation, **(b)** *S. pasteurii*-mediated precipitation, **(c)** *P. fluorescens*-mediated precipitation, and **(d)** purified EPS-mediated precipitation. Diffractograms show phase identification. Variations in peak intensity and broadening, and phase composition, reflect differences in mineral crystallinity and phase distribution depending on the bacterial/EPS interface.

**Table 1:** Phase content (wt%) determined by XRD (semi)quantitative analysis of precipitates.

Phase	ICDD card number	Abiotic	*S. pasteurii*	*P. fluorescens*	EPS
Apatite-type calcium phosphate†	–	61	–	–	–
Hydroxyapatite	86–1203	–	100	92	37
Brushite	72–713	13	–	–	–
Monetite	70–359	26	–	8	–
Calcium hydrogen phosphate hydrate (monetite-like)	26–1056	–	–	–	63

The simultaneous presence of brushite, monetite and apatite-type calcium phosphate demonstrates that the abiotic system produced a mixed crystalline calcium phosphate assemblage under the experimental conditions. The persistence of dicalcium phosphate phases alongside the apatite-type component is consistent with incomplete transformation toward apatite.

In contrast to the abiotic control, the *S. pasteurii*-mediated system exhibited hydroxyapatite as the only crystalline phase detected (100 wt% of the identified crystalline fraction) (Figure 4b). No intermediate dicalcium phosphate phases, including brushite or monetite, were detected within the limits of the XRD analysis. The absence of detected dicalcium phosphate phases is consistent with more complete phase transformation toward apatite under these conditions. Diffraction peaks were sharp and well defined, consistent with comparatively advanced crystallographic development. Relative to the abiotic system, the *S. pasteurii*-mediated system therefore exhibited a more HAp-dominated crystalline assemblage without detectable dicalcium phosphate phases.

The *P. fluorescens*-mediated system showed hydroxyapatite as the dominant identified crystalline phase (~92 wt%), accompanied by a minor monetite fraction (~8 wt%) (Figure 4c). Brushite was not detected. Compared with *S. pasteurii*, the persistence of a small monetite fraction is consistent with less complete conversion of the detected crystalline calcium phosphate phases to HAp. Nevertheless, the predominance of HAp demonstrates substantial phase maturation relative to the abiotic control. The diffraction peaks exhibited slightly greater broadening than those of the *S. pasteurii*-mediated precipitate, suggesting differences in crystallite size and/or structural ordering.

The EPS treatment displayed a markedly different phase composition. A calcium hydrogen phosphate hydrate (monetite-like) phase constituted the dominant identified crystalline fraction (approximately 63 wt%), whereas hydroxyapatite represented approximately 37 wt% (Figure 4d). This phase assemblage indicates that the tested EPS fraction supported calcium phosphate precipitation, but conversion to crystalline apatite remained incomplete during the five-day incubation period. The persistence of the precursor-like phase may reflect ion binding, transport or hydration conditions within the polymeric matrix; however, its stabilization by EPS cannot be established conclusively from the present experimental design. Compared with the intact-cell treatments, the EPS treatment therefore contained a smaller proportion of crystalline hydroxyapatite.

All phase proportions in Table 1 refer to the identified crystalline phases only and do not account for amorphous calcium phosphate (section 2.5). Consequently, detection of hydroxyapatite as the only crystalline phase in the *S. pasteurii* system does not establish phase purity of the bulk precipitate, and this qualification also applies when interpreting the surface-area and dissolution data that follow.

Combining the phase proportions in Table 1 with the phosphate removed from solution allows the mineral mass in each system to be estimated independently of the gravimetric measurement. The resulting values are approximately 1.54 g L^−1^ for *S. pasteurii,* 1.54 g L^−1^ for *P. fluorescens*, 1.21 g L^−1^ for the EPS-mediated system and 0.82 g L^−1^ for the abiotic system. The close agreement between the calculated mineral mass and the gravimetric value for the abiotic system (0.84 g L^−1^) supports the internal consistency of the solution-chemistry and phase analyses. The greater gravimetrically recovered mass in the biologically mediated systems may additionally reflect organic material associated with the recovered precipitates.

### 3.4 FESEM morphology overview: four-system comparison

FESEM analysis revealed pronounced morphological differences among the four precipitation systems, highlighting the distinct roles of bacterial cells and extracellular interfaces in regulating calcium phosphate mineral formation (Figures 5, 6).

**Figure 5:**
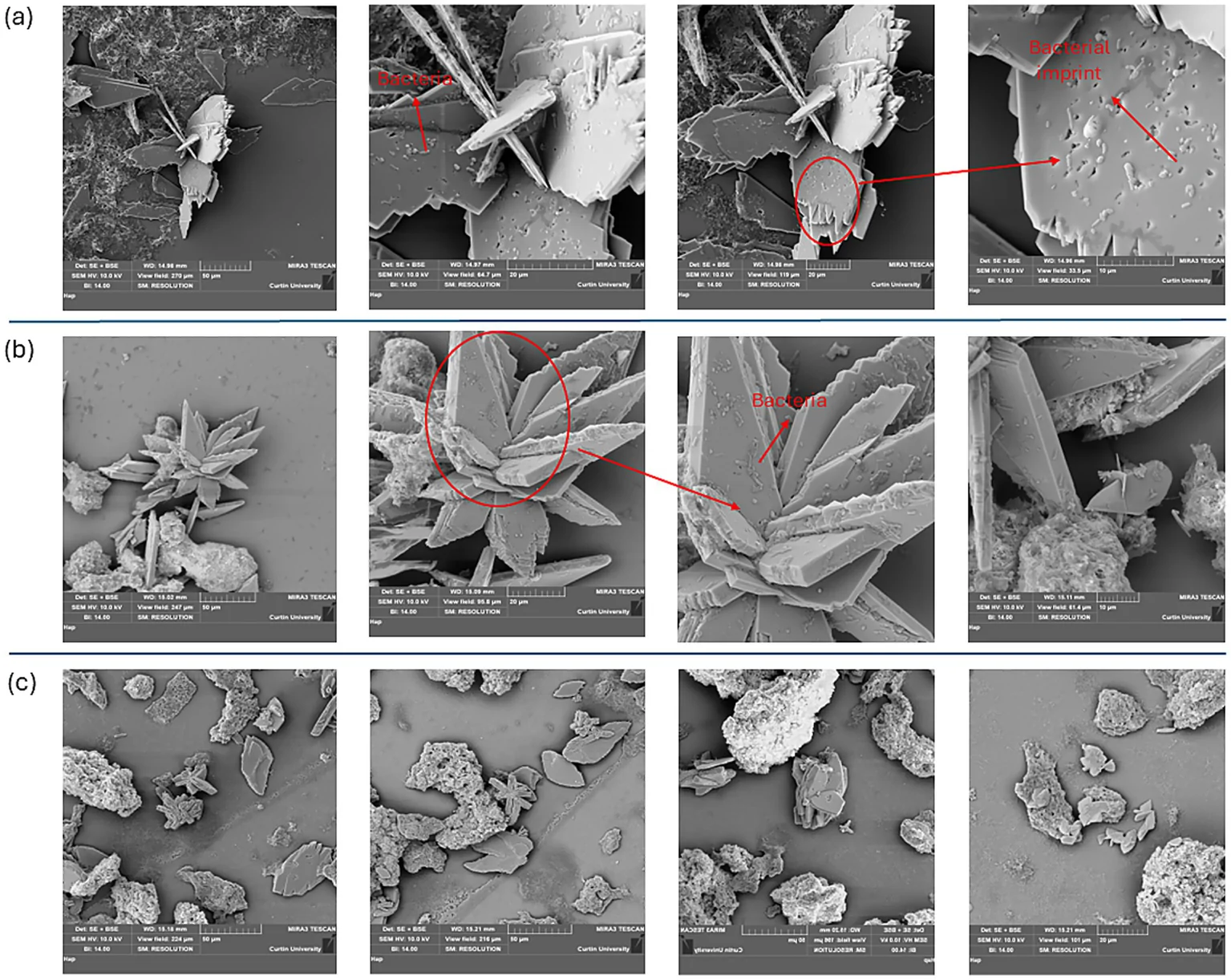
FESEM images of calcium phosphate precipitates formed via different bacterial interfaces: **(a)** *S. pasteurii*-mediated precipitation, **(b)** *P. fluorescens*-mediated precipitation, and **(c)** precipitation induced by isolated EPS.

**Figure 6:**
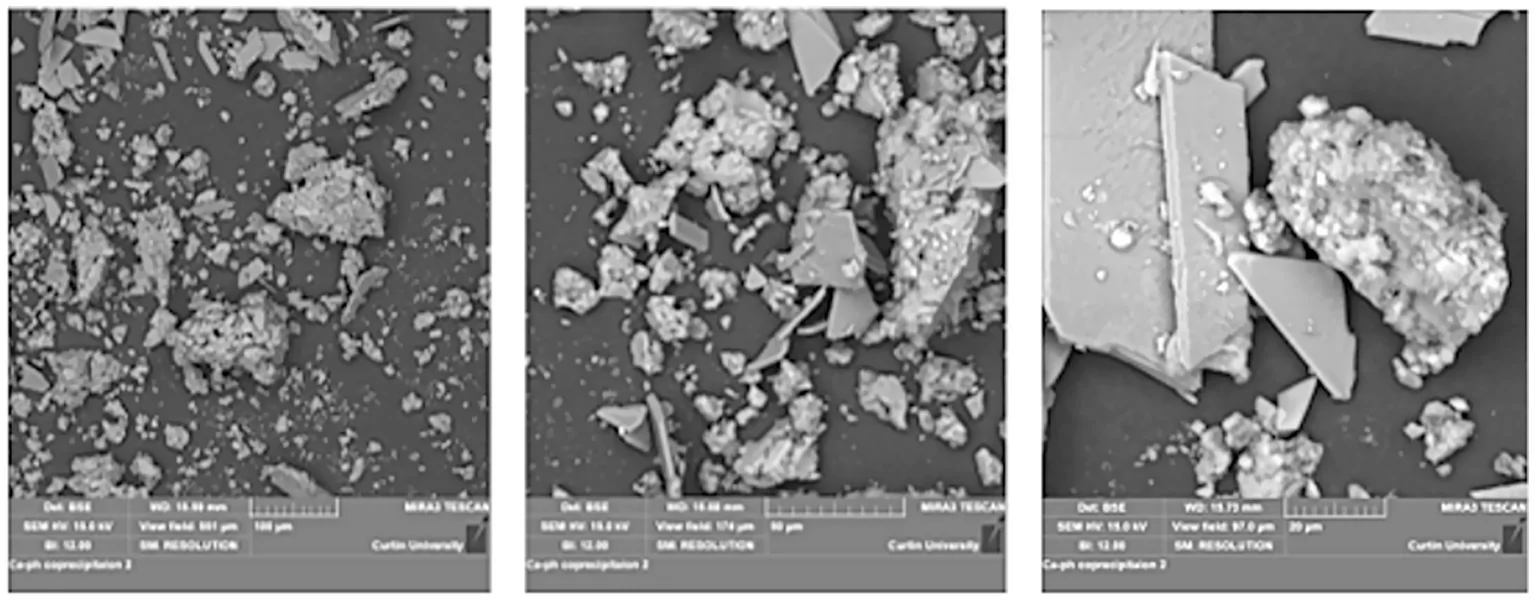
FESEM images of abiotic calcium phosphate precipitates at increasing magnifications.

The *S. pasteurii*-mediated samples (Figure 5a) were characterized by the formation of large, well-developed plate-like crystals, typically ranging from approximately 2–80 μm in lateral dimension, with some aggregates exceeding this size. The crystals exhibited sharp, well-defined faceted edges and relatively smooth surfaces, indicative of advanced crystallographic development. Layered stacking and intergrowth of lamellae, along with plate-like structures, were frequently observed, suggesting successive crystal growth episodes under sustained supersaturation. Several crystal surfaces also exhibited localized cell-sized pits or impressions with dimensions comparable to bacterial cells. These features are consistent with a possible contribution from cell-associated interfacial interactions during mineral formation; however, their presence alone does not establish the location or mechanism of initial mineral nucleation.

In contrast, *P. fluorescens*-mediated precipitation (Figure 5b) produced dense, radially organized aggregates with a characteristic flower-like morphology. The crystals formed radially arranged plate-like lamellae that radiated outward from central growth points, forming clusters approximately 10–40 μm in diameter. Individual lamellae were generally thinner and more densely arranged than those observed in *S. pasteurii* samples. Many crystals appeared partially embedded within surrounding material, suggesting the presence of extracellular polymers during mineral formation. Compared with *S. pasteurii*, the *P. fluorescens* samples exhibited a higher degree of aggregation and structural intergrowth. Crystal faceting remained visible; however, crystal boundaries were often less distinct, reflecting the influence of organic interfaces on crystal growth.

Precipitation in the EPS treatment (Figure 5c) resulted in markedly different morphologies. The EPS-mediated system displayed smaller, irregular aggregates composed of fine particulate material and fragmented plate-like domains, typically below 20 μm in size. The crystals lacked the extensive lateral growth and pronounced faceting observed in the whole-cell systems. Instead, aggregates appeared granular and loosely structured, with a greater abundance of fine particulate material. No clear evidence of large, continuous lamellar stacking was observed, indicating reduced crystal organization and structural development.

Collectively, the whole-cell treatments produced larger and more structurally developed calcium phosphate crystals than the isolated EPS treatment. *S. pasteurii*-mediated precipitation produced larger, well-faceted plate-like crystals, whereas *P. fluorescens*-mediated precipitation generated more radially organized and matrix-associated aggregates. The observed cell-sized surface features and mineral architectures are consistent with a possible contribution from cell–mineral interfacial interactions and localized ion accumulation; however, FESEM morphology alone does not establish the location or mechanism of initial mineral nucleation.

Abiotic precipitates (Figure 6) exhibited a highly heterogeneous morphology characterized by irregularly distributed aggregates and irregular crystalline fragments. FESEM observations at increasing magnifications revealed a mixture of large plate-like crystals with sharp edges and fractured surfaces coexisting with fine granular clusters and loosely packed particulate material. Crystal sizes varied widely, ranging from sub-10 μm particles to larger angular plates approximately 40–60 μm in lateral dimension. Although the plate-like structures appeared relatively smooth and well-defined, they lacked consistent orientation, organized stacking, or radial symmetry. Crystal surfaces were frequently associated with randomly attached granular material and compact agglomerates, consistent with rapid physicochemical nucleation followed by irregular crystal growth and secondary aggregation. The broad particle size distribution and fragmented morphology indicated mineral formation governed primarily by physicochemical precipitation.

Supplementary non-viable-cell controls were additionally examined to determine whether calcium phosphate mineralization persisted following cell inactivation (Supplementary Figure S1). PFA-fixed *S. pasteurii* and *P. fluorescens* treatments exhibited only modest changes in bulk pH during the five-day incubation, while qualitative XRD identified HAp in precipitates recovered from both fixed-cell treatments. FESEM observations further demonstrated extensive crystalline mineral formation. Thus, HAp formation persisted in the presence of non-viable cellular interfaces, indicating that continued cellular viability was not an absolute requirement for mineral precipitation under the conditions investigated.

### 3.5 Surface area and pore structure of calcium phosphate precipitates

BET surface-area analysis and BJH pore characterization were performed across the four experimental systems (Table 2). XRD analysis showed that hydroxyapatite dominated the identified crystalline fraction of the *S. pasteurii*- and *P. fluorescens*-mediated precipitates, whereas the abiotic and EPS-mediated systems contained substantial proportions of non-apatitic calcium phosphate phases. Accordingly, the BET and BJH measurements were interpreted together with the XRD and FESEM observations rather than as independent indicators of mineral structure. Because the XRD phase proportions describe only the identified crystalline fraction, any amorphous and organic components present in the recovered material may also have contributed to the measured surface characteristics.

**Table 2:** BET surface area and pore characteristics of calcium phosphate precipitates from all experimental systems.

Sample	Dominant phase	BET surface area (m^2^ g^−1^)	BJH cumulative pore volume (cm^3^ g^−1^)	BJH desorption average pore width (nm)
*S. pasteurii*	Hydroxyapatite	14.30	0.059	12.08
*P. fluorescens*	Hydroxyapatite	14.42	0.049	16.93
EPS-mediated	Mixed-phase calcium phosphate	56.20	0.122	11.96
Abiotic	Mixed-phase calcium phosphate	12.21	0.041	13.13

The EPS-mediated precipitate exhibited the highest measured BET specific surface area (~56 m^2^ g^−1^) and the largest BJH cumulative pore volume (0.122 cm^3^ g^−1^). In comparison, the abiotic precipitate exhibited a BET surface area of approximately 12 m^2^ g^−1^ and a cumulative pore volume of 0.041 cm^3^ g^−1^. The *S. pasteurii*- and *P. fluorescens-*mediated precipitates exhibited comparable BET surface areas of approximately 14 m^2^ g^−1^, with cumulative pore volumes of 0.059 and 0.049 cm^3^ g^−1^, respectively. Residual EPS within the recovered EPS-mediated precipitate may have contributed to its comparatively high measured surface area and pore volume.

BJH pore-size analysis further showed differences in average pore width across the recovered precipitates, ranging from 11.96 to 16.93 nm (Table 2). The EPS-mediated precipitate combined the highest measured surface area and cumulative pore volume with the smallest average pore width. These measurements describe the textural characteristics of the recovered precipitates but should be considered together with differences in crystalline phase assemblage, morphology and possible organic content among the experimental systems.

The BET/BJH measurements represent single determinations performed on pooled material from each experimental system (section 2.7) and therefore do not provide an estimate of between-incubation or analytical variability. In addition, several of the precipitates exhibited relatively low specific surface areas. The BET values are consequently presented as preliminary, descriptive estimates, and small numerical differences between treatments are not interpreted quantitatively.

### 3.6 Acid dissolution: quantifying HAp durability

The acid dissolution experiment revealed clear differences in the stability of calcium phosphate precipitates produced under the four experimental conditions (Figure 7). All samples exhibited progressive mass loss during exposure to the pH 4.0 acetate buffer; however, the extent of dissolution differed substantially among treatments. Because the recovered precipitates differed in mineral phase composition and textural characteristics, dissolution was evaluated on a mass basis to compare the acid resistance of the as-formed materials.

**Figure 7:**
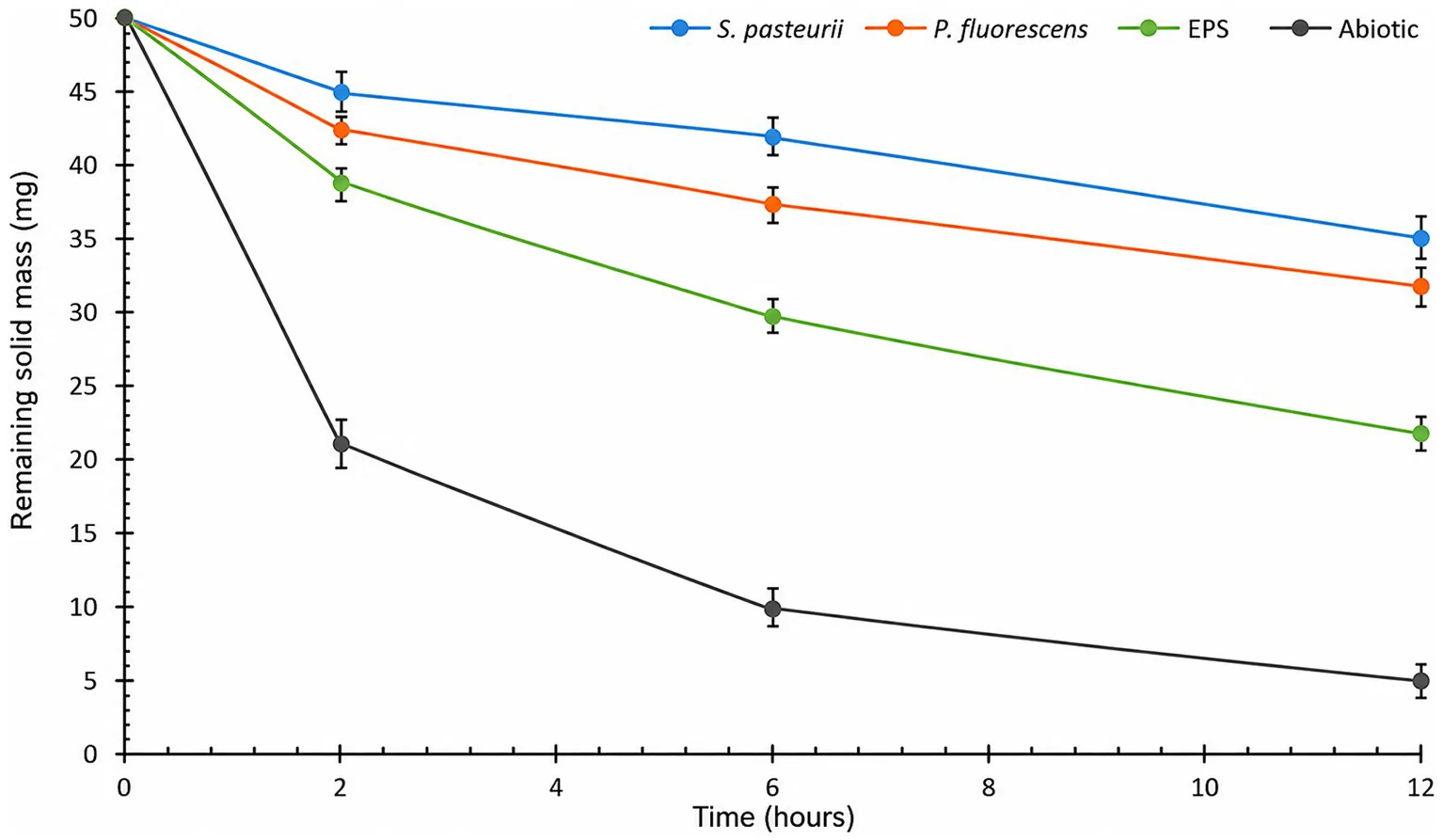
Acid dissolution behavior of calcium phosphate precipitates from all four experimental systems at pH 4.0. Data represent mean ± SD of three replicate dissolution assays on pooled precipitate (*n* = 3).

Overall, the extent of dissolution followed the trend: abiotic > EPS-mediated *> P. fluorescens* > *S. pasteurii* (Figure 7). After 12 h, the abiotic precipitate retained approximately 10% of its initial mass, whereas the EPS-mediated precipitate retained 22 mg (44%). The two bacterial precipitates exhibited greater mass retention, with the *P. fluorescens*-mediated precipitate retaining 32 mg (64%) and the *S. pasteurii*-mediated precipitate retaining 35 mg (70%). Thus, among the materials examined, the *S. pasteurii*-mediated precipitate exhibited the greatest mass retention under the acidic conditions tested.

The dissolution behavior across the four systems was not explained by the preliminary BET surface-area estimates alone. Despite exhibiting the highest measured BET surface area (~56 m^2^ g^−1^), the EPS-mediated precipitate dissolved more slowly than the abiotic control, which exhibited a substantially lower measured surface area (~12 m^2^ g^−1^). The dissolution trends were therefore more closely associated with differences in crystalline phase assemblage than with the preliminary BET surface-area estimates. This observation is consistent with the established differences in acid solubility among calcium phosphate phases, with less stable calcium phosphate precursor phases generally exhibiting greater solubility than hydroxyapatite under acidic conditions (Wang and Nancollas, 2008). Consistent with this interpretation, XRD showed substantial proportions of non-apatitic calcium phosphate phases in the abiotic and EPS-mediated precipitates, whereas HAp dominated the identified crystalline fractions of the bacterial precipitates.

Because the two HAp-rich bacterial precipitates exhibited comparable preliminary BET surface-area estimates (~14 m^2^ g^−1^ for both *S. pasteurii* and *P. fluorescens*), the modest difference in their acid-dissolution behavior cannot be attributed confidently to differences in measured surface area. Surface-area-normalized dissolution rates were therefore not used to infer quantitative differences between the bacterial treatments. Instead, the BET measurements are considered descriptively alongside differences in crystalline phase assemblage and mineral morphology identified by XRD and FESEM.

Overall, the mass-retention results demonstrate that the HAp-rich bacterial precipitates exhibited greater resistance to acid dissolution than the more compositionally heterogeneous abiotic and EPS-mediated precipitates under the conditions tested. These findings are consistent with the greater acid stability associated with apatite-rich calcium phosphate materials (Wang and Nancollas, 2008), while recognizing that differences in phase composition, morphology, surface characteristics and associated organic material may all contribute to the dissolution behavior of the recovered precipitates.

Overall, the four experimental systems exhibited distinct precipitation kinetics, crystalline phase assemblages, mineral morphologies and acid-dissolution behavior. Whole-cell *S. pasteurii* and *P. fluorescens* treatments produced HAp-rich precipitates with greater acid resistance than the EPS-mediated and abiotic systems, while the isolated EPS treatment produced a more precursor-rich mineral assemblage. Supplementary PFA-fixed-cell experiments further demonstrated that HAp precipitation could persist in the absence of continued cellular viability. Together, these observations provide the experimental basis for distinguishing viability-dependent changes in solution chemistry from viability-independent interfacial contributions to calcium phosphate mineralization, as considered further in the Discussion.

## 4 Discussion

Calcium orthophosphates are salts of tribasic phosphoric acid (H₃PO₄) and comprise a wide range of crystalline and amorphous phases that differ in composition, hydration state and stability (Dorozhkin, 2007; Wang and Nancollas, 2008). Their precipitation can proceed through multistep pathways consistent with Ostwald’s rule of stages, in which metastable phases may form before more stable phases develop, although the sequence depends on the experimental conditions (Wang and Nancollas, 2008). Rather than forming directly as hydroxyapatite [HAp; Ca₁₀(PO₄)₆(OH)₂], calcium phosphate may form through pathways involving amorphous calcium phosphate (ACP), brushite (CaHPO₄·2H₂O), monetite (CaHPO₄) and octacalcium phosphate [OCP; Ca₈H₂(PO₄)₆·5H₂O] before apatite develops (Ibsen et al., 2016). The relative abundance of these phases is influenced by pH, Ca/P ratio, phosphate speciation, ion activity, hydration state and the presence of organic interfaces capable of binding and reorganizing ions (Štulajterová and Medvecký, 2008). Consequently, the endpoint phase assemblage reflects both bulk solution chemistry and the local interfacial environment in which mineralization occurs (Kumar et al., 1999; Yanovska et al., 2012).

ACP-mediated pathways involving nanoscale calcium phosphate clusters have also been proposed during apatite formation (Lotsari et al., 2018). Although ACP was not directly detected in the present study, transient ACP formation cannot be excluded. It is therefore considered here only as a possible, unconfirmed precursor rather than an experimentally identified phase (Combes and Rey, 2010; Mancardi et al., 2017).

Within this framework, the differences in endpoint phase composition among the treatments are consistent with an influence of bacterial cells and extracellular polymers on calcium phosphate formation. XRD revealed distinct phase assemblages in the abiotic, isolated-EPS and whole-cell systems, but the endpoint patterns do not by themselves establish the transformation pathway or separate interfacial effects from changes in solution chemistry. Mineralization performance was therefore considered using three distinct outcomes: the rate and extent of Ca^2+^ and phosphate removal, the predominance of crystalline HAp in the recovered precipitates, and resistance of the recovered solid to acid dissolution. These outcomes were not interchangeable; rapid ion removal or high recovered mass did not necessarily correspond to formation of a more mature or durable apatite-rich precipitate.

The recovery of mineral from the abiotic control demonstrates that calcium phosphate precipitation occurred without a biological interface. The abiotic precipitate comprised apatite-type calcium phosphate (61 wt%), brushite (13 wt%) and monetite (26 wt%). The apatite contribution was initially fitted as separate hydroxyapatite and chlorapatite components; however, because these structurally related apatite endmembers have strongly overlapped diffraction patterns, they could not be reliably resolved as independently crystallized phases from the present XRD data and were therefore considered collectively as apatite-type calcium phosphate. The coexistence of apatite-type material with brushite and monetite is consistent with incomplete transformation of dicalcium phosphate phases toward apatite under the experimental conditions (Kumar et al., 1999; da Silva et al., 2001). Nucleation in the abiotic system could have occurred through solution-based calcium-phosphate association or heterogeneously on available non-biological surfaces (Dey et al., 2010; Habraken et al., 2013).

The transient decrease in bulk pH observed during early abiotic precipitation is consistent with a net acidifying contribution from mineral formation. Apatite formation from HPO₄^2−^-containing solutions can release H^+^, whereas phosphate buffering and interfacial proton exchange can modify the measured bulk response (Wang and Nancollas, 2008). However, local interfacial pH and proton fluxes were not measured directly, and the bulk pH profile does not establish that proton release was responsible for persistence of brushite or monetite. The abiotic results therefore establish spontaneous precipitation and incomplete apatite predominance under the tested bulk chemical conditions without assigning a unique transformation mechanism.

The isolated-EPS system also produced a mixed crystalline assemblage, with a calcium hydrogen phosphate hydrate (monetite-like) phase representing approximately 63 wt% of the identified crystalline fraction and HAp approximately 37 wt%. Here, “EPS” refers specifically to the fraction isolated from 48 h *P. fluorescens* cultures grown in LB medium. This treatment represents a cell-free polymeric-interface model rather than a species-matched EPS comparison or a reconstruction of the extracellular material produced during the five-day mineralization experiment. Accordingly, the EPS-only treatment demonstrates that the isolated polymeric fraction supported calcium phosphate precipitation, but its contribution cannot be directly subtracted from the whole-cell response.

By contrast, both whole-cell systems produced HAp-rich crystalline assemblages. The *S. pasteurii* system exhibited HAp as the only crystalline phase detected, whereas *P. fluorescens* produced predominantly HAp with a minor monetite fraction. These findings show an association between intact bacterial cells and greater conversion toward crystalline HAp relative to the abiotic and isolated-EPS systems. They are consistent with a contribution from cell-associated interfacial chemistry and cell-envelope architecture to mineral development, but do not by themselves establish the molecular mechanism or location of initial nucleation.

Although the primary whole-cell experiments were conducted with metabolically active cells, supplementary PFA-fixed non-viable-cell controls provide additional insight into the requirement for continued cellular activity (Supplementary Figure S1). No colony formation was detected from the fixed preparations under the tested growth conditions, while both fixed-cell treatments produced crystalline HAp-containing precipitates. Bulk pH changed only modestly during the five-day incubation. These observations demonstrate that continued cellular viability was not an absolute requirement for HAp precipitation under the conditions investigated and are consistent with a contribution from viability-independent physicochemical interactions at bacterial interfaces. Nevertheless, PFA fixation cross-links cellular components and may alter the accessibility or reactivity of surface functional groups; fixed cells therefore cannot be assumed to be chemically equivalent to native viable-cell surfaces. In addition, the supplementary XRD analysis was qualitative and does not establish equivalent phase proportions or phase purity between live- and fixed-cell treatments. The fixed-cell controls consequently support, but do not directly prove, a passive cell-surface nucleation mechanism and do not exclude metabolic contributions in the live-cell systems.

Bacterial surfaces can provide sites for heterogeneous nucleation because negatively charged functional groups can associate with divalent cations and influence local ion distributions (Douglas and Beveridge, 1998; Beveridge et al., 2003; Hoffmann et al., 2021). In Gram-positive bacteria such as *S. pasteurii*, carboxyl groups within peptidoglycan and phosphate-containing wall teichoic acids contribute to negative surface charge (Navarre and Schneewind, 1999; Rohde, 2019). *S. pasteurii* exhibited the least negative zeta potential of the biological interfaces (approximately −25 mV), while FTIR showed carboxylate- and phosphate-associated features. Wall teichoic acids are densely distributed within the Gram-positive cell wall and can provide fixed anionic sites capable of interacting with divalent cations (Swoboda et al., 2010; Schneewind and Missiakas, 2014). This organization provides a plausible interfacial explanation for the greater conversion toward crystalline HAp observed in the *S. pasteurii* system despite its less negative measured zeta potential.

In contrast, the more negative zeta potential of *P. fluorescens* (approximately −38 mV) is consistent with the chemistry of the Gram-negative envelope, which includes an outer membrane rich in phospholipids and lipopolysaccharides containing phosphorylated and carboxylated groups (Beveridge, 1999; Di Lorenzo et al., 2021). Its FTIR spectrum also showed strong carboxylate- and phosphate-associated features. These characteristics are consistent with substantial capacity for interfacial Ca^2+^ association and may contribute to the rapid ion removal observed in this system. However, the present measurements do not establish a direct causal relationship between zeta potential, ion-binding density and the subsequent mineral phase assemblage. Moreover, the zeta potentials were measured in 0.5 mM KCl at pH 7.4 and therefore provide a comparative index of interfacial charge rather than the effective electrostatic potential operating in the mineralization medium containing 16 mM Ca^2+^. The two species were also cultivated in different growth media, which may influence envelope composition and extracellular polymer production.

The isolated EPS fraction exhibited the most negative zeta potential (approximately −49 mV) and FTIR features associated with carboxyl- and phosphate-containing groups. These observations are consistent with a hydrated polymeric interface capable of interacting strongly with dissolved cations. Previous studies have shown that EPS can concentrate ions, modify hydration and transport, and influence the persistence of amorphous or metastable mineral precursors (Obst et al., 2009; Gutierrez et al., 2012; Decho, 2010; Zhang et al., 2021). Such processes provide plausible explanations for the precursor-rich crystalline assemblage observed in the isolated-EPS treatment, although the present experiments do not distinguish among these mechanisms directly.

The precipitation kinetics further demonstrate that ion removal and mineral maturation were not directly coupled. All biological treatments showed greater Ca^2+^ and phosphate removal than the abiotic control, with the most rapid removal observed for *P. fluorescens*. However, the fastest ion removal did not correspond to the greatest predominance of crystalline HAp. *S. pasteurii*, despite exhibiting the least negative zeta potential of the biological interfaces, produced HAp as the only crystalline phase detected. These observations indicate that the magnitude of measured surface charge alone does not predict apatite maturation and suggest that additional interfacial characteristics, including the chemical identity, accessibility and spatial organization of ion-binding functional groups, may contribute to the observed differences in mineral phase evolution.

The pH profiles likewise reflect the net balance among mineral precipitation, phosphate buffering, proton exchange at interfaces and, in the viable whole-cell treatments, possible residual metabolic activity. Calcium phosphate precipitation can release H^+^ depending on the aqueous phosphate species incorporated into the mineral (Wang and Nancollas, 2008). For example, HAp formation from hydrogen phosphate can be represented by the balanced reaction (Chan et al., 2023):

$$10{\mathrm{Ca}}^{2+}+\mathrm{6HPO}{₄}^{2-}+\mathrm{2H}₂O\to {\mathrm{Ca}}_{10}{\left(\mathrm{PO}₄\right)}_{6}(\mathrm{OH})₂+{\mathrm{8H}}^{+}.$$

Formation of dicalcium phosphate phases from the same aqueous phosphate species can involve substantially less proton release. The measured pH profiles should therefore be interpreted as the combined outcome of precipitation chemistry, aqueous buffering and biological/interfacial processes rather than as a direct measure of mineral precipitation alone. The modest increase in bulk pH in the live whole-cell treatments suggests that cellular processes may have partly offset precipitation-associated proton production. Although the medium contained neither urea nor an organic phosphate substrate, it contained 2 g L^−1^ yeast extract and could therefore support residual cellular activity. Importantly, HAp-containing precipitates also formed in the PFA-fixed treatments despite only modest bulk pH changes (Supplementary Figure S1), showing that pronounced bulk alkalinization was not required for HAp formation under the tested conditions. Because local interfacial pH was not measured and PFA fixation modifies cellular chemistry, the relative contributions of metabolism and surface physicochemical interactions cannot be quantified from the present experiments.

The minor monetite fraction detected in the *P. fluorescens* precipitate may partly reflect the influence of cell-associated EPS. The isolated *P. fluorescens*-derived EPS treatment produced a more precursor-rich crystalline assemblage, with a calcium hydrogen phosphate hydrate (monetite-like) phase dominating the identified crystalline fraction. This makes EPS-mediated mineralization a plausible contributor to the more polyphasic character of the *P. fluorescens* precipitate relative to *S. pasteurii*. However, EPS production was not quantified in the whole-cell treatments, and the isolated EPS was recovered from LB-grown cultures before mineralization. Its contribution therefore cannot be distinguished conclusively from effects associated with the Gram-negative cell envelope or residual cellular activity. Direct testing would require complementary experiments such as EPS-depleted *P. fluorescens* cells and addition of isolated EPS to the *S. pasteurii* system under matched conditions.

Taken together, the surface-chemistry and kinetic results show that the magnitude of measured surface charge alone did not predict the final crystalline phase assemblage. The more negatively charged *P. fluorescens* and isolated-EPS interfaces were associated with rapid ion removal, whereas *S. pasteurii*, despite its less negative zeta potential, exhibited the greatest conversion toward crystalline HAp. This contrast suggests that mineralization behavior depends on additional interfacial properties beyond net surface charge. The present data are consistent with contributions from the chemical identity, accessibility and spatial organization of ion-binding functional groups, but do not establish which individual property is the dominant mechanistic determinant.

FESEM observations further showed that the experimental interfaces were associated with distinct mineral architectures. The abiotic system produced heterogeneous aggregates comprising angular plate-like crystals together with finer particulate material. The *S. pasteurii* system produced large, faceted plate-like crystals, with cell-sized pits or impressions on some crystal surfaces. These features are consistent with a possible contribution from cell-associated interfacial interactions during mineral growth, but do not by themselves demonstrate that initial nucleation occurred directly on the cell envelope. The *P. fluorescens* system generated smaller, radially organized flower-like aggregates, many associated with matrix-like material, whereas the isolated-EPS system produced smaller and less structurally organized aggregates. Together with the XRD results, these morphological differences are consistent with distinct interfacial environments during mineralization; however, FESEM morphology alone cannot determine the molecular mechanism of nucleation or subsequent phase transformation.

Despite the marked differences in crystalline phase composition and morphology, the *S. pasteurii*- and *P. fluorescens*-mediated precipitates exhibited comparable preliminary BET surface-area estimates of approximately 14 m^2^ g^−1^. The EPS-mediated precipitate exhibited a higher measured surface area (~56 m^2^ g^−1^), whereas the abiotic precipitate exhibited the lowest value (~12 m^2^ g^−1^). Because these measurements were single determinations on pooled material and several samples exhibited relatively low specific surface areas, they are interpreted as preliminary, descriptive estimates rather than precise quantitative differences among treatments. The comparable BET estimates for the two bacterial precipitates indicate that their different acid-dissolution behavior cannot be confidently attributed to measured surface-area differences. BET therefore provides useful textural context but should be considered together with phase assemblage, morphology and associated organic material.

The acid dissolution experiment provided a functional comparison of mineral durability across the four systems. After 12 h at pH 4.0, the abiotic precipitate retained only approximately 10% of its initial mass, the EPS-mediated precipitate retained approximately 44%, and the whole-cell precipitates retained approximately 64–70%. These trends are consistent with established differences in the acid stability of calcium phosphate phases, with non-apatitic precursor phases generally exhibiting greater solubility than HAp under acidic conditions (Wang and Nancollas, 2008). Consistent with this interpretation, the abiotic and EPS-mediated precipitates contained substantial proportions of non-apatitic crystalline phases, whereas HAp dominated the identified crystalline fractions of the whole-cell precipitates. Importantly, the dissolution trends were not explained by the preliminary BET surface-area estimates alone. Because the precipitates also differed in phase assemblage, morphology and associated organic material, the present experiment does not isolate any single property as the sole determinant of acid stability.

These findings have implications for biotechnological applications involving microbially induced calcium phosphate mineralization. In contexts where long-term mineral stability is important, including acid mine drainage treatment, subsurface biobarriers, remediation of phosphate-rich environments and durable mineral-based materials, precipitation yield alone may not adequately describe mineralization performance. The crystalline phase assemblage and resistance of the resulting mineral to dissolution should also be considered when selecting or engineering microbial mineralization systems.

## 5 Conclusion

The present study demonstrates that bacterial interfaces are associated with distinct calcium phosphate mineralization outcomes under identical initial solution chemistry. Mineralization performance was evaluated using three complementary criteria: (i) the rate and extent of Ca^2+^ and phosphate removal, (ii) the predominance of crystalline HAp in the recovered precipitate, and (iii) resistance of the resulting mineral to acid dissolution. These criteria were not directly coupled, demonstrating that a system exhibiting faster ion removal did not necessarily produce the most HAp-dominated or acid-resistant mineral.

The major findings of this study are as follows:

- The degree of surface charge alone did not predict mineralization outcome. *P. fluorescens* achieved the most rapid removal of Ca^2+^ and phosphate from solution, whereas *S. pasteurii*, despite exhibiting the least negative zeta potential among the biological interfaces, produced HAp as the only crystalline phase detected. Thus, *P. fluorescens* performed most strongly with respect to ion-removal kinetics, while *S. pasteurii* showed the greatest predominance of crystalline HAp. These findings demonstrate that rapid ion sequestration and apatite maturation are distinct outcomes and are consistent with a contribution from interfacial properties beyond net surface charge, including the chemical identity, accessibility and spatial organization of ion-binding functional groups.
- The isolated *P. fluorescens*-derived EPS supported calcium phosphate precipitation but produced a more precursor-rich crystalline assemblage than the intact-cell treatments. The persistence of a minor monetite fraction in the *P. fluorescens* whole-cell precipitate may partly reflect the influence of cell-associated EPS. However, because EPS production was not quantified in the whole-cell treatments, its contribution cannot be separated conclusively from effects associated with the Gram-negative cell envelope or residual cellular activity.
- Continued cellular viability was not an absolute requirement for HAp precipitation under the conditions investigated. Supplementary PFA-fixed non-viable *S. pasteurii* and *P. fluorescens* treatments produced HAp-containing crystalline precipitates despite loss of detectable colony-forming ability and only modest changes in bulk pH. These controls are consistent with a contribution from viability-independent physicochemical interactions at bacterial interfaces, although PFA fixation may alter surface chemistry and therefore does not establish passive cell-surface nucleation as the sole mechanism.
- The results have implications for the design of microbially induced calcium phosphate mineralization systems. For applications including acid mine drainage remediation, subsurface biobarriers, treatment of phosphate-rich environments and mineral-based materials, mineralization performance should not be assessed from precipitation yield alone. Ion-removal efficiency, crystalline phase development and resistance to dissolution represent distinct performance criteria that should be considered according to the intended application.

Future work should examine whether these interfacial effects persist across a wider range of bacterial species, solution conditions and timescales. Experiments using EPS-depleted cells, species-matched isolated EPS and complementary surface-characterization approaches would help resolve the relative contributions of cell-envelope chemistry and extracellular polymers to calcium phosphate mineralization.

## Data Availability

The original contributions presented in the study are included in the article/Supplementary material, further inquiries can be directed to the corresponding author.
