# Supplementary material for: Bacterial interfaces and surface chemistry shape hydroxyapatite mineral architecture during microbially induced calcium phosphate precipitation
Source: Front Microbiol. 2026 Oct 26;17:1949480. doi: 10.3389/fmicb.2026.1949480 (PMC13745221; doi:10.3389/fmicb.2026.1949480)
Supplement: Supplementary file 1 [file Supplementary_file_1.docx]

**Supplementary Data**

**S1. Supplementary Materials and Methods**

Additional mineralisation experiments were conducted using paraformaldehyde (PFA)-fixed cells of *S. pasteurii* and *P. fluorescens* to determine whether calcium phosphate mineral formation could occur in the absence of continued cellular viability. PFA fixation was selected as an aldehyde-based inactivation approach because it preserves overall bacterial cell morphology and retains the extracellular polymeric layer, which is largely removed by glutaraldehyde fixation, although some surface filaments may be lost (Chao and Zhang, 2011).

Cultures of both species were grown as described in Section 2.1 and harvested at mid-exponential phase (OD₆₀₀ ≈ 0.5). Cells were recovered by centrifugation, washed with sterile phosphate-free buffer, and subsequently resuspended in 4% (w/v) PFA. Cell suspensions were fixed for 30 min at room temperature. Following fixation, cells were recovered by centrifugation and washed twice with sterile phosphate-free buffer to remove residual PFA.

Loss of colony-forming ability following PFA fixation was experimentally verified by inoculating the treated cells onto their corresponding growth media. PFA-fixed *S. pasteurii* cells were inoculated onto YAT agar, whereas PFA-fixed *P. fluorescens* cells were inoculated onto LB agar. No detectable colony formation was observed following incubation under the respective growth conditions, confirming that the PFA-fixed preparations had lost colony-forming ability under the conditions tested.

Following confirmation of inactivation, PFA-fixed cells were introduced into the calcium phosphate precipitation medium at an initial cellular biomass equivalent to that used in the corresponding live-cell treatments. Mineralisation assays were conducted using the same medium composition, working volume, incubation temperature, agitation rate, and experimental duration described for the live-cell mineralisation experiments. Each non-viable-cell treatment was conducted in triplicate using three independent flasks (n = 3) within a single experimental run.

Bulk pH was monitored over the five-day incubation period. At the completion of mineralisation, precipitates were recovered and characterised by scanning electron microscopy (SEM) and X-ray diffraction (XRD). XRD patterns were used for qualitative phase identification to determine the presence of hydroxyapatite (HAp); RIR-based semi-quantitative phase analysis was not performed for these supplementary control samples.

**S2. Supplementary Results**

Only modest changes in bulk pH occurred in the PFA-fixed treatments over the five-day mineralisation period (Fig. S1). The PFA-fixed *S. pasteurii* treatment varied from pH 8.0 on Day 1 to 8.3 on Day 5, and the PFA-fixed *P. fluorescens* treatment similarly remained within a narrow range, from pH 8.0 on Day 1 to 8.2 on Day 5. Both non-viable-cell treatments therefore remained mildly alkaline throughout the experiment.

The limited variation in bulk pH is consistent with the absence of continued metabolic modification of the medium. Mineral precipitation and HAp formation were nevertheless detected in these treatments, indicating that large viability-dependent changes in bulk pH were not a prerequisite for HAp formation under the experimental conditions.

XRD analysis confirmed the formation of a crystalline HAp phase in the PFA-fixed non-viable-cell treatments (Fig. S1). In both treatments the experimental patterns reproduced the principal reflections of the HAp reference pattern, including the characteristic group in the approximately 31–33° 2θ region together with HAp-associated reflections at lower and higher diffraction angles. HAp was therefore identified as the principal crystalline phase in both precipitates. Minor reflections that could not be assigned to HAp were also present, and quantitative phase analysis was not performed for this series; these data therefore establish phase identification rather than phase purity.

The occurrence of HAp in the non-viable-cell treatments, together with the comparatively stable bulk pH, indicates that continued bacterial viability was not an absolute requirement for HAp precipitation in this supersaturated calcium phosphate system. The result is consistent with a contribution from viability-independent physicochemical processes in systems containing bacterial interfaces. Because PFA fixation cross-links cellular components and can alter the accessibility or reactivity of surface functional groups, however, the fixed-cell experiments should not be interpreted as demonstrating chemical equivalence between live and fixed bacterial surfaces.

SEM examination revealed extensive crystalline precipitation in the PFA-fixed non-viable-cell treatments (Fig. S1). The recovered products displayed prominent plate-like to bladed crystals, including aggregated and locally radiating architectures, together with finer particulate deposits on and around the larger mineral structures. These observations provide morphological evidence that substantial mineral precipitation occurred in the absence of viable cells.

SEM morphology alone was not used to assign mineral phase identity; phase identification was based on XRD. Taken together, the SEM and XRD observations show that crystalline HAp-containing precipitates formed in the presence of non-viable bacterial material. This finding is consistent with, but does not by itself prove, a contribution from passive cell-associated heterogeneous nucleation.

**Fig. S1**. Mineralisation experiments with PFA-fixed non-viable cells of (a) S. pasteurii and (b) P. fluorescens. For each treatment the panels show, from left to right: FESEM images, XRD patterns of the recovered precipitates and bulk pH during the incubation (mean ± SD, n = 3).

**Supplementary references**

Chao, Y., Zhang, T., 2011. Optimization of fixation methods for observation of bacterial cell morphology and surface ultrastructures by atomic force microscopy. Applied Microbiology and Biotechnology 92 (2), 381–392. https://doi.org/10.1007/s00253-011-3551-5
